# A Data-Driven Prediction Method for an Early Warning of Coccidiosis in Intensive Livestock Systems: A Preliminary Study

**DOI:** 10.3390/ani10040747

**Published:** 2020-04-24

**Authors:** Federica Borgonovo, Valentina Ferrante, Guido Grilli, Riccardo Pascuzzo, Simone Vantini, Marcella Guarino

**Affiliations:** 1 Department of Environmental Science and Policy, Università degli Studi di Milano, 20133 Milan, Italy; federica.borgonovo@unimi.it (F.B.); marcella.guarino@unimi.it (M.G.); 2 Department of Veterinary Medicine, Università degli Studi di Milano, 20133 Milan, Italy; guido.grilli@unimi.it; 3 Neuroradiology Unit, Fondazione IRCCS Istituto Neurologico Carlo Besta, 20133 Milan, Italy; riccardo.pascuzzo@istituto-besta.it; 4 MOX-Department of Mathematics, Politecnico di Milano, 20133 Milan, Italy; simone.vantini@polimi.it

**Keywords:** Poultry, early warning system, VOCs, coccidiosis, data-driven machine learning algorithm

## Abstract

**Simple Summary:**

The development of new methods, able to promptly detect the onset of the infection, is highly important to control parasitic infections of the intestinal tract (coccidiosis) in poultry. The early detection of this disease would reduce the use of anticoccidial and antimicrobials drugs, thus lowering the risk of antibiotic resistance. A data-driven machine learning algorithm was built to relate air quality data to the time of enteric disorders. The results show that this procedure has great potential to be used as a rapid technique to detect coccidiosis.

**Abstract:**

Coccidiosis is still one of the major parasitic infections in poultry. It is caused by protozoa of the genus *Eimeria*, which cause concrete economic losses due to malabsorption, bad feed conversion rate, reduced weight gain, and increased mortality. The greatest damage is registered in commercial poultry farms because birds are reared together in large numbers and high densities. Unfortunately, these enteric pathologies are not preventable, and their diagnosis is only available when the disease is full-blown. For these reasons, the preventive use of anticoccidials—some of these with antimicrobial action—is a common practice in intensive farming, and this type of management leads to the release of drugs in the environment which contributes to the phenomenon of antibiotic resistance. Due to the high relevance of this issue, the early detection of any health problem is of great importance to improve animal welfare in intensive farming. Three prototypes, previously calibrated and adjusted, were developed and tested in three different experimental poultry farms in order to evaluate whether the system was able to identify the coccidia infection in intensive poultry farms early. For this purpose, a data-driven machine learning algorithm was built, and specific critical values of volatile organic compounds (VOCs) were found to be associated with abnormal levels of oocystis count at an early stage of the disease. This result supports the feasibility of building an automatic data-driven machine learning algorithm for an early warning of coccidiosis.

## 1. Introduction

In intensive broiler farms, enteric disorders represent a major health issue and are caused by bacteria, viruses, and parasites. One of the most common enteric disorders in broiler farming is coccidiosis. The economic significance of this enteric disease is attributed to reduced animal production (higher feed conversion, growth depression, and increased mortality) and the costs concerning treatment and prevention [1,2]. Moreover, coccidiosis can greatly affect animal welfare.

Coccidiosis is caused by a group of protozoa of the *Eimeriidae* family that infect the animal bowel. These parasites, also known as coccidia, are generally present in poultry-raising operations. Moreover, they are host-specific and can parasitize specific parts of the intestine, where they cause inflammation and several degrees of injury. According to the most common broiler farming systems, the environmental conditions, i.e., the high density and large number of animal reared, might promote coccidiosis development [3]. Today, prevention and control systems of coccidiosis are based on, (1) chemoprophylaxis (anticoccidial products or anticoccidials in the feed) and (2) vaccination, along with hygienic measures and improved farm management [2,4,5]. Chemoprophylaxis is by far the most popular. In particular, two groups of anticoccidial are generally used: ionophoroes antibiotics, or ‘ionophores’; and synthetically produced drugs, or ‛chemicals’. This clashes with the public’s concern regarding the use of drugs in intensive farming [6].

Coccidiosis is still a major problem and this is mainly due to its difficult diagnosis, also taking into account that the typical parasitological methods of diagnosis are labour intensive and therefore costly. Moreover, these techniques require expertise and are performed by only a few laboratories [4].

The main technique is oocyst per gram (OPG) count in faeces/litter, which allows the identification of different *Eimeria* species based on the oocystis morphology. Another method is lesion scoring, which is an interpretation based on visible lesions provoked by *Eimeria* in the different intestinal tracts in dead/culled animals [7].

For these reasons, it is crucial to develop new vaccines, diagnostics, novel therapies, and management strategies [8] that are able to promptly react to the infestation in the early stages. New sensitive and ultra-rapid diagnostic methods have been explored to evaluate the possibility of diagnosing pathologies in livestock and humans. These new techniques are based on the identification of volatile organic compounds (VOCs) produced by pathogens, host–pathogen interactions, and biochemical pathways [9]. Indeed, it has been shown that pathophysiological responses to infections, toxins, or endogenous metabolic pathway perturbations affect the qualitative and quantitative composition of VOCs that are present in the biological materials of humans and animals (blood, breath, stool, sweat, skin, urine, and vaginal fluids) [10]. In particular, odor and air quality from livestock may indicate animals’ health, and the enteric disease may be characterized by different chemical odor properties [11,12,13].

In our previous study, we observed a strong relationship between air quality data and the number of oocysts present in different broiler houses, suggesting that air sample analysis may detect the presence of coccidia infection in animals reared in commercial poultry farms [12]. In this scenario, a highly sensitive and specific sensor-based device was developed to collect and analyze the air in the farm. The purpose of this pilot study was to evaluate the applicability of a data-driven prediction algorithm for an early warning of enteropathies in intensive broiler farms. Such a method is easily repeatable, cost and labor efficient, and can identify changes in VOCs compound profile in the air inside sheds. The use of this innovative approach might provide a fast and reliable early warning for this pathology.

## 2. Materials and Methods

The trial was carried out in three intensive poultry farms located in northern Italy and lasted six months during autumn, winter, and spring. We monitored three complete life cycles of broilers in two different farms (Farm 1 and 2) and two cycles in Farm 3. Three identical devices were installed in the farms.

In all of the farms, Ross 308 males were reared and, based on the EU Council Directive on Broiler Welfare (Directive 2007/43/EC), the stocking density was 33 kg/m^2^ [14].

All farms were equipped with forced ventilation by negative-pressure systems, automatic systems to monitor the environmental parameters, and litters of wood shavings. In order to classify the dust level in the broiler shed, the dust sheet test was performed [15].

### 2.1. Ethical Statement

Ethical approval was not necessary as the devices work without any direct contact with poultry.

### 2.2. Poultry Diet

The feeding program was divided into three periods: (1) the animals were fed with a starter in the 1st 10 days; (2) for the following 14–16 days, the animals were fed with grower; finally, (3) the animals were fed with finishing feed starting from 25 days of age until slaughter.

The poultry’s nutrition management was similar for all housing during the experiment and formulated according to the specification provided for Ross 308. Furthermore, management programs were designed to prevent coccidiosis development, resulting in better gut health and feed utilization by animals. Animals were fed with the same diet in all three farms, with the addition of anticoccidials in the ration according to this scheme: Halofugine from 0 to 11 days of age, Salinomicin from 12 to 22 days of age, and Monensin from 23 days of age to 7 days before slaughter.

### 2.3. Collection of Faeces Samples and VOCs Analysis

**Figure 1:**
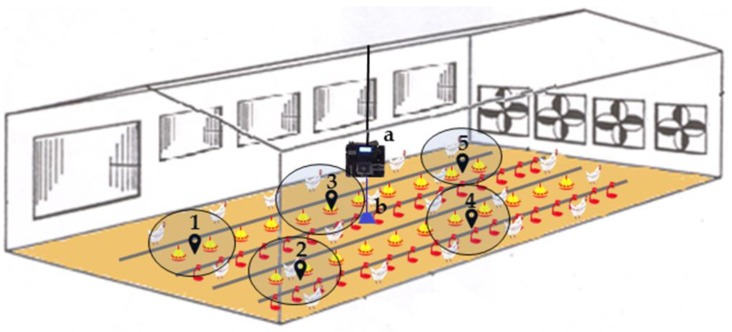
Illustration of the faeces sampling zones (1–5), the position of the device within the shed (**a**), and the air collection point (**b**).

Faeces samples were collected on a weekly basis in 5 different zones inside each shed monitored (Figure 1). Each sample includes 50 faeces (25 from cecum). Faeces samples were collected in vials to perform oocystis count at the laboratory of avian pathology of the University of Milan, in accordance with the Mc Master method [16]. The number of oocystis, expressed as oocystis g^−1^ [faeces] (OPG), was used as a Gold Standard to indicate the health status of broilers (infected/not infected).

In one shed per farm, an analysis of volatile organic compounds (VOCs) was performed on air samples collected by a device based on a metal oxide semiconductor (MOS) [12], which was placed in the middle of the building (see Figure 1). The device did not directly measure the presence of coccidia but rather the presence of VOCs, which are well correlated with animal health [12].

The air was continuously drawn into the device throughout the production cycle by means of a polytetrafluoroethylene tube (Teflon). The air was sampled at 40 cm above the floor and drawn into a small chamber consisting of an electronic non-specific sensor array that is sensitive to a wide range of VOCs. Changes in the electrical characteristics of the sensors are due to the superficial reaction resulting from gas absorption. The electrical signals produced from the sensors were recorded every 10 s for further analysis. The system is based on a patented technology (International Publication Number: WO2017/212437).

### 2.4. Instrument Calibration

The devices were calibrated and adjusted before using them in the farms to ensure a precise and reliable measurement. On the one hand, calibration allows compensation for sensor wear and it is carried out at least twice a year by a specialized technician. On the other hand, adjustment aims to improve instrument accuracy and precision through a complete reset of the sensors, instrument, and data analysis software, and must be carried out to ensure a “zero” reference before positioning the instrument on-site.

### 2.5. Data Processing

In order to focus the analysis on early warning for coccidiosis, we selected the sensors’ data collected from the start of each broiler life cycle until the time at which the maximum oocyst number was recorded. Data related to the last part of the life cycle were not considered informative for early warning and were excluded from the analysis. Selected data were then split into training and test sets for each farm: the first two cycles of Farm 1 and 2, and the first cycle of Farm 3 composed the training set, while the remaining cycle of each farm was assigned to the test set (cycle 3 of Farm 1 and 2 and cycle 2 of Farm 3). The sensors’ data of each cycle in the training set were processed separately using principal component analysis (PCA) to obtain a reduced representation of the data. For each farm, data in the test set were projected on the space generated by the principal components (PCs) of the corresponding training set. Moreover, we performed a monotone cubic spline interpolation of the sampled oocystis number of each cycle to obtain a continuous representation in the considered time domain, assuming a monotone increase in the number of oocystis in this time window, as also suggested by other authors [17].

To achieve our aim, we applied a k-nearest neighbors algorithm (k-nn) to compute the probability that the number of oocystis exceeded a predetermined threshold at each time point, based on the scores of the first two PCs. The number k of nearest neighbors considered in the final model was optimized separately for each farm through a leave-one-group-out cross-validation procedure, where we excluded the data of one day at the time.

The accuracy of the prediction was computed on the test set for each farm by calculating the area under the receiver operating characteristic (ROC) curve.

The analysis was carried out in R 3.6.1 (R Core Team, Vienna, Austria), using the “kknn” package for the k-nearest neighbors algorithm.

## 3. Results

### 3.1. Environmental Parameters

The temperature and the relative humidity of all farms were regularly monitored to ensure a uniform environment throughout the cycles. The humidity was 60–70% during the first 3 days and less than 60% thereafter. The temperature ranged from 27 to 30 °C in the first week and progressively decreased as the cycle advanced, reaching 20 °C after 27 days of age. Then, the temperature remained constant around 18–20 °C throughout the rest of the cycle. The ventilation rate (m^3^/hr) in all three farms ranged from 0.080 to 1.969–2.258 m^3^/hr according to the animals’ size during the growing period.

The main contaminants (dust, ammonia, carbon dioxide, and carbon monoxide) were kept within legal limits in all three farms and at all times. Furthermore, using good management practices, the dust particle concentration was kept to a minimum (the mean score of the dust sheet test was 1, corresponding to minimal evidence of dust).

### 3.2. Production Data

Ross 308 is a bird with fast growth, excellent conversion, and good bird performance. The slaughter age of the broiler was similar in all three farms and ranged from 45 to 51 days in Farm 1, from 43 to 46 days in Farm 2, and finally, from 44 to 52 days in Farm 3.

The feed conversion rate was associated with the slaughter age in all three farms. In particular, an increase in the feed conversion rate was observed in parallel with an increase in the slaughter age. The feed conversion rate showed values that ranged from 1.61, corresponding to 43 days of slaughter, to 1.78, corresponding to 52 days of slaughter.

The average daily weight gain, as for the feed conversion rate, increased proportionally to the slaughter age of broilers. In particular, the average daily weight gain ranged from 74.46 g, corresponding to 43 days of slaughter, to 79.80 g, corresponding to 52 days of slaughter.

The slaughter weight, considering all three farms, ranged from 3.2 to 4.1 kg. All three farms had low cumulative mortality, ranging from 2.01 to 4.31%.

### 3.3. Oocyst Count (Gold Standard) and Threshold

From each set of faeces, collected weekly in each farm, five aliquots of sample were taken and analysed. The number of oocystis in faecal samples acquired at variable days are displayed in Table 1. These results were obtained by averaging the number of oocystis observed in the five aliquots collected. In all cycles, the OPG count increased from the beginning to the fourth/fifth week and then decreased; the highest values were found at week 4 or 5 and reached peaks of 68,500 in Farm 1.

The measured numbers of oocystis were used to interpolate the oocyst count at every time point between days 0 and 28 or 35, to associate an oocyst number to each recorded signal of the sensors.

**Figure 2:**
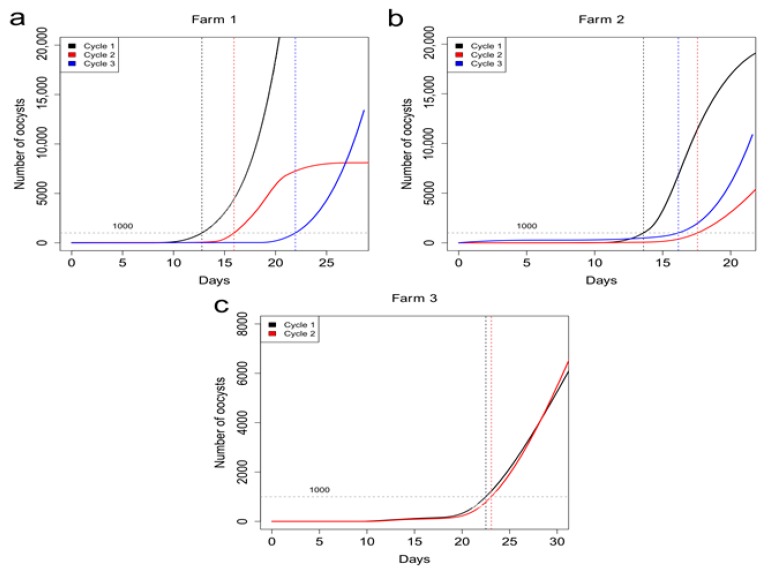
The number of oocystis interpolated from the oocyst count for each cycle and farm. The horizontal grey line refers to 1000 oocystis level. Farm 1 (**a**); Farm 2 (**b**); Farm 3 (**c**).

From numerous tests that have previously been carried out (unpublished data), we have noticed that 1000 oocystis allow for the opportunity to intervene with natural products for a possible therapy with natural substances (polyphenols, essential oils) before subclinical or clinical coccidiosis is established. The numerical progression of the oocystis present in the faeces over time is illustrated in Figure 2, which shows that all curves grow quite abruptly after the threshold of 1000 oocystis.

### 3.4. Principal Components Analysis

**Figure 3:**
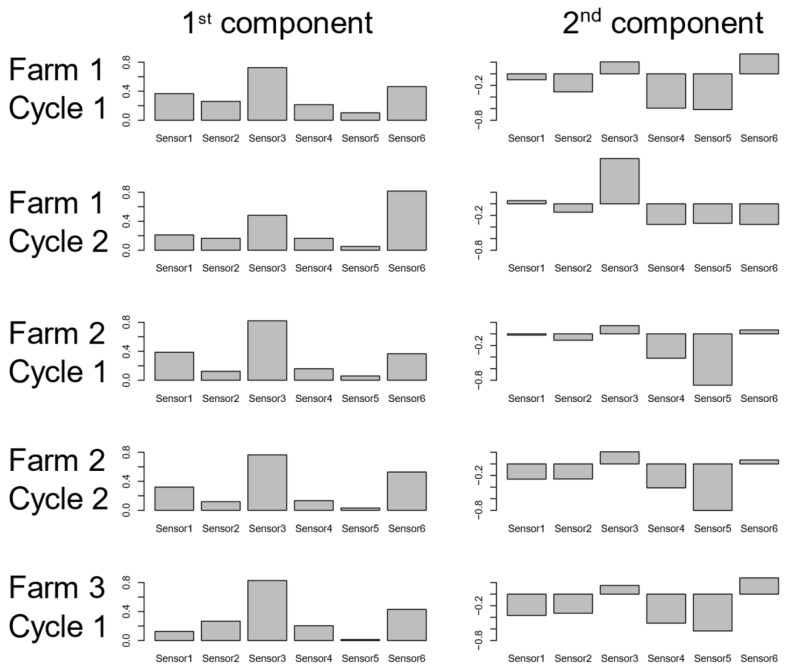
Barplot of the loadings of the first two principal components for each cycle of the three farms in the training set.

Each broiler farm was analyzed separately. The PCA components (PCs) describe directions of data variation and are influenced by the original features. Such influences, or loadings, are the weights used to linearly combine the sensors’ responses, and they are associated to the importance of each input in generating the PCs. From the comparisons between the PCA loadings of the cycles in the training set (Figure 3), there were similarities in the combinations used to generate the principal components among cycles.

The first component was mostly influenced by inputs 3 and 6, and the second component was a contrast of inputs 3 and 6 against the others, especially input 5. These similarities among cycles are important findings in terms of the reproducibility of the study. Percentages of the total variance explained by the first two PCs were above 96% for the cycles in the training set in all farms (Table 2).

### 3.5. Infection Prediction

**Figure 4:**
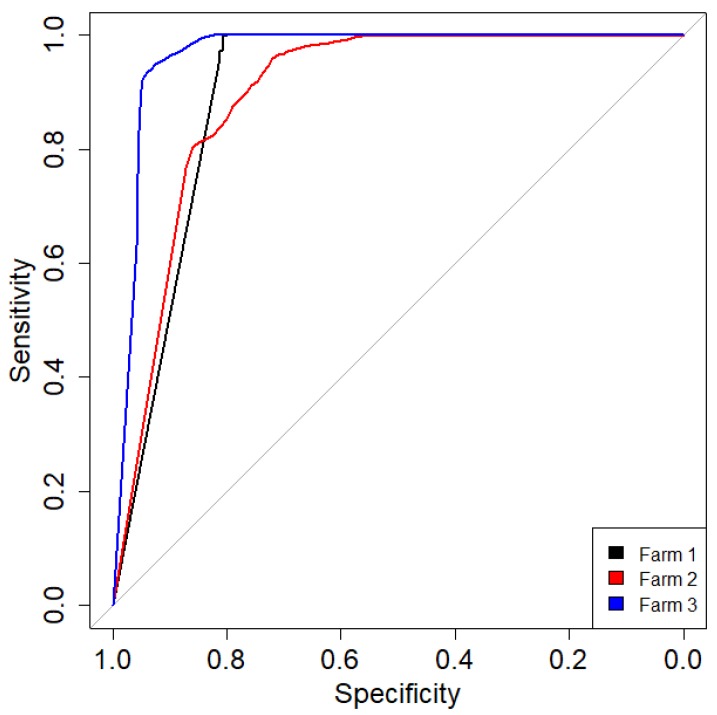
Receiver operating characteristic (ROC) curves for the test data of the three farms.

To evaluate whether the system was able to early identify the coccidia infection in intensive poultry farms, a k-nn was trained on the scores of the first two PCs and tested on unseen data from the same farms. Figure 4 shows the resulting ROC curves of the k-nn algorithm when applied to the test cycles of the three farms: the area under the curve (AUC) was high for all farms (Table 3).

## 4. Discussion

According to the gold standard (oocystis count), k-nn shows excellent capability to identify the 1000 level of oocystis in the test set, providing evidence for the feasibility of a system for an early warning of enteropathies in intensive broiler farms.

The PCA results suggest that similarities among the two–dimensional representation were present among the cycles, highlighting how a change in VOCs’ composition, and thus in odour properties, can be used as an indicator of oocystis increment. Indeed, odour modification is influenced by various factors, such as litter conditions, environment, microbial activity, and management practices [9,12,18]. By infesting the intestinal mucosa, *Eimeria* spp. cause lesions in the gut. These intestinal pathogens influence the intestinal microflora, and major shifts in the microbiome can be observed following coccidial infection in the cecum, as reported by Martynova-Van Kley et al. [19]. Microbiota in the gastro-intestinal tract then move to the litter, causing a variation in the VOCs’ composition in the air [1,19] that can be detected by our device and then identified by the proposed algorithm.

This has enabled the reliable identification of a possible threshold (i.e., 1000 oocystis) corresponding to an early significant increment of the number of oocystis. Such a threshold was accurately identified in every tested cycle, supporting the robustness and the repeatability of the study in different broiler houses. The optimal k can influence the predictive ability of the model; when k is small, the number of data points that influence the prediction is accordingly small and forces the classifier to be “more blind” to the general data distribution, decreasing the prediction’s bias. Conversely, a large k reduces the impact of variance due to a random error by providing a more smoothed output [20]. A trade-off between these two extremes is usually solved by a cross-validation, as in this study, in order to identify the optimal k.

## 5. Limitations

It was not possible to change the normal farm management and avoid economic damage to the farmers. To overcome this problem, we have chosen three farms with similar management, and similar results have been obtained. Consequently, further research should include the analysis of several farms with different management systems to assess the generalizability of these results in other scenarios.

Moreover, only three farms have been studied due to the limited number of devices available. Only one cycle per farm was used to test the model performances.

In the future, it will be necessary to increase the number of cycles tested to support the repeatability of this study.

## 6. Conclusions

This study shows that the proposed method is able to identify the coccidia infection in intensive poultry farms early. Moreover, its application in livestock farming could be advantageous. Air analysis revealed that the discrimination was related to a significant change in the number of oocystis at a very early stage, as confirmed by the gold standard. This method is well suited for the methodologies and the goals of precision livestock farming, and this device might provide a real-time monitoring system to improve animal health by using an alert notification when the infection arises so that the veterinarian may take immediate action.

In conclusion, the results of this study support the feasibility of building an automatic data-driven machine learning algorithm for an early warning of coccidiosis. In future studies, further information should be considered to improve the performance of this model, such as the temporal dependency of data and the broiler house effect.

## Figures and Tables

**Table 1:** Oocyst count in all the three farms during the production cycle.

Farms	Cycle Number	Cycle Days					
		Week 1	Week 2	Week 3	Week 4	Week 5	Week 6
Farm 1	1st	0	275	9500	68,500	2850	1250
	2nd	0	125	6800	8100	11,000	10,075
	3rd	0	25	750	12,800	12,175	9625
Farm 2	1st	0	25	9700	19,900	1350	1200
	2nd	0	50	3075	15,250	5150	1075
	3rd	350	525	8825	N/A	N/A	N/A
Farm 3	1st	0	75	325	3300	8000	5150
	2nd	0	75	375	3900	10,000	5850

N/A: data not available.

**Table 2:** Percentages of variances explained by the first two principal components for the cycles in the training set.

Broiler House	Cycle	Variance Explained (%)
1	1	97.0
	2	97.8
2	1	96.4
	2	97.6
3	1	96.1

**Table 3:** K-nearest neighbors parameter (k) computed and optimized on the training set and the area under the curve (AUC) computed on the test set.

Broiler House	Optimal k	AUC
1	7	0.902
2	35	0.897
3	70	0.967
